# An integrative framework for mapping the psychological landscape of risk perception

**DOI:** 10.1038/s41598-024-59189-y

**Published:** 2024-05-14

**Authors:** Sarah C. Jenkins, Robert F. Lachlan, Magda Osman

**Affiliations:** 1 Centre for Decision Research, Leeds Business School, University of Leeds, Leeds, Yorkshire LS2 9JT UK; 2 Department of Psychology, Royal Holloway University of London, Egham, Surrey TW20 0EX UK; 3 Judge Business School, University of Cambridge, Trumpington Street, Cambridge, CB2 1AG UK

**Keywords:** Psychology, Human behaviour

## Abstract

We vary greatly in our perception of risk, not just because of differences between risks themselves, but also because of individual, contextual and cultural differences too. To better understand and predict responses to risk, we need to (a) integrate these components, combining approaches from different psychological disciplines and (b) also consider risk tolerance – how individuals trade-off between risks and benefits. We therefore developed an ICONS (individual, contextual, cognitive, social) framework; using it across two empirical studies (n = 4228) to examine how individuals perceive and respond to the quotidian risks associated with consumer products. Three dimensions underlined risk perceptions: benefits, dread and individual responsibility. Risk tolerance was typically predicted by interactions between individual (demographic, cultural worldview, personality) and contextual (product type/category, harm information) factors. In turn, perceived dread, benefits and individual differences shaped how likely participants were to communicate risk information. Our results demonstrate for the first time how the *interaction* between individual, cognitive (risk tolerance, intensity), contextual, and social (risk communication) factors is key to understanding and predicting risk perceptions. Together, our findings help explain why societal responses to risks are often difficult to predict and have implications for the spread, and amplification, of risk information.

## Introduction

The study of risk perception has provided insight into how we assess and respond to societal hazards, often focusing on those with catastrophic potential, such as nuclear power, terrorism and climate change^1–3^. But risk is also something that we confront daily in almost every decision we make, yet despite its ubiquity, this aspect of risk perception has been comparatively neglected by the literature. One context where risk is inherent is in consumer product choices: “should I buy the newly advertised combined washer-dryer-iron appliance, or should I buy the traditional ones separately?” or “should I allow my child to use a e-scooter to ride to school?” Advances in technology mean that new products are regularly coming to market, the risks of which consumers must assess. Although the risk associated with any one consumer item is small, their combined risk across society is significant. In spite of decades of research and government interventions, injuries and deaths caused by home, leisure and school accidents (including those involving consumer products) were by far the biggest cause of non-fatal injuries across the European Union between 2009 and 2018^4^. Despite this, in general, such risks are tolerated – that is the trade-off between perceived risk and benefits is weighted towards the latter. Furthermore, such decisions (whether to buy a product or whether to use it) are made very frequently, though little is understood with regards to the contribution that perceptions and tolerances of risk make to day-to-day consumer decisions^5^. Here, we argue a different, more holistic perspective is required to fully understand risk perceptions and their relation to risk tolerance, particularly given the nature of such quotidian risks.

Previous perspectives such as the cognitive or ‘psychometric’ approach originated from the observation that the public’s risk perceptions did not always reflect technical assessments of ‘objective’ risk (based on probability of hazard × severity of harm). Of course, in day-to-day life, individuals typically assess risks with very limited access to reliable probability information, meaning perceptions and judgements are formed in relation to other, non-technical factors^6^. Instead, individuals use a range of heuristics to process risk information, such as severity, familiarity and controllability, which influence how much risk is perceived^7^. Given such assessments take place with limited access to objective information, we can expect (and indeed find) that people sometimes overestimate and sometimes underestimate the dangers they face^8^.

However, a focus on the factors driving risk perceptions is only one part of the psychological landscape; attention should also be paid to the *function* of risk perceptions – that is, how an individual weighs up risks against benefits to inform their tolerance of risks when making decisions to act based on their appraisals. This can be neatly illustrated for quotidian hazards, where risks are relatively low, the cognitive integration of risk with benefits is especially important. Whilst the role of benefits in shaping risk perceptions has been previously highlighted^9–11^, here we argue that they also have ramifications for how much risk individuals are willing to *tolerate* – essentially the trade-off between risks and benefits. The finance literature has long since recognised the trade-off between risk and return and relatedly, the notion of risk tolerance (often referred to as risk preference – “the tendency to be attracted or repelled by alternatives that are perceived as risky” p.142^12^). Although such a mechanism has been explored previously in psychological research on risk^13^, we use a richer, more holistic measurement of perceived risk than previously used in order to calculate risk tolerance. From this, our novel proposal, which is empirically investigated, is that understanding risk perceptions requires a complementary understanding of *risk tolerance*, because risk tolerance is likely to directly inform an individual’s actions beyond their perceptions of risk. That is, an individual may perceive a high risk for a particular item, but the effect of this is moderated by the level of benefits, with high benefits outweighing the perceived risks, reflective of risk tolerance. An analogous situation is the interplay between risk assessment and risk management, in which a risk is identified and quantified, and a decision still required as to what action should be taken. This new framing of risk tolerance represents an explanation for why risk perceptions have not always been found to consistently predict behaviour^14^.

In focusing largely on cognitive factors, the psychometric approach has neglected the role of individual, social, and cultural factors in shaping risk judgements^15^, and relatedly that of trust^16^, and how they might explain the variance between people in their perceptions of risk. To address this, other studies have focused on person-specific factors such as demographics and personality. Socio-demographic characteristics such as age and gender have also been consistently, if weakly, associated with risk perceptions, with lower risk perceptions seen for young people and/or males^17–19^. Many traits have been identified as correlated with risk perceptions, albeit also relatively weakly, including risk propensity^20–22^, risk preference^23^, risk sensitivity^24^ and openness to new experiences^25^. Yet attitudes towards risk are not always stable, with differences observed across contexts^21^ argued to reflect the limited explanatory contribution of individual characteristics in shaping risk perceptions. Crucially though, their (perceived) limited importance may be a result of the narrow focus of previous research, rather than accurately reflecting the contribution of individual characteristics.

A third group of explanatory factors explore the role that cultural biases (reflecting values and beliefs – ‘worldviews’) play in generating inter-individual differences in risk perception. The cultural theory (CT) of risk^26–28^ argues that risk is socially constructed and is dependent on cultural context (for a review see Ref.^29^). Specifically, cultural biases and social relations influence risk perceptions, with increased risk perceived when hazards threaten one’s worldview^30^. However, worldviews explain only limited variance in risk perceptions^31^ and their predictive value has not always been consistently demonstrated^15,32–34^.

These advances in understanding of risk perceptions still remain limited because they typically investigate risk from within silos: taking one perspective while excluding other contributing factors. As such, existing literature is a challenging landscape to draw firm insights and recommendations from; at best the findings are disparate and at worst, highly contradictory. It is for this reason that to fully answer the question of “why might one individual perceive a risk as particularly severe, and another not?”, beginning at the individual level to integrate across individual, cognitive, contextual and social factors is a practical solution, before moving on to population level dynamics^35^. For example, the risk of a vaccine specifically administered to children versus adults might have a cognitive component related to the recipients’ risk of illness, but this may be perceived differently by men and women, or parents versus non-parents, or those with hierarchical versus egalitarian worldviews. Without considering these factors together, one risks mistakenly asserting that certain factors are not of great import in the formation of risk perceptions, when in fact they are, but rather in conjunction with other factors. The current study was thus designed to test the relative contribution of these factors, and their potential interaction, as well as considering the downstream effects on the public’s risk tolerance.

In fact, this interplay also exposes another layer of factors that have been largely ignored in risk perception studies: what determines the day-to-day communication of risk experiences to others. While it is necessary in the process of risk assessment and risk management to have an agreed mechanism for communicating risk between experts, it is less clear which factors inform the basis for at an individual level. The social amplification framework [SARF]^36^ was developed as a framework for exploring how social context could influence how a risk is perceived, understood, communicated – potentially lead to amplification or attenuation of the risk within a society. However, the SARF initially focused on the role of the mass media, but the advent of new communication channels (including social media) presents new challenges for understanding risk communication^37^. More pertinently, SARF focuses of on communication at a societal level, rather than fully exploring the nuances of individual perceptions and what these might mean for information dissemination. Ultimately, risk dissemination does not necessarily mean social amplification^38^, which SARF fails to capture, paving the way for the current, individually based approach.

**Figure 1:**
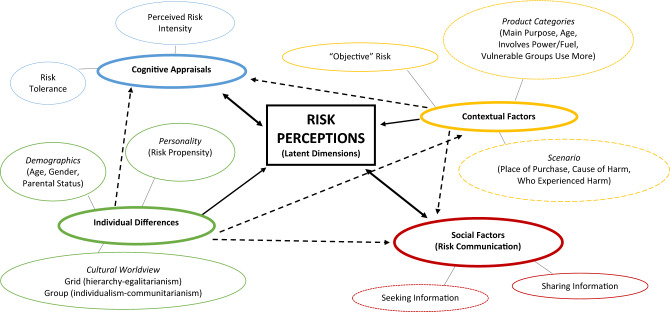
The individual, contextual, cognitive, social (ICONS) framework. An overview of our integrative approach to investigating risk perceptions, in which the factors identified link and interact with each other to shape risk perceptions. Factors surrounded by continuous lines were investigated in both Studies 1 and 2, and those surround by dotted [dashed] lines investigated only in Study 1 [Study 2]. Given its inclusion in the technical risk assessment process, “objective” risk is included for completeness but was not investigated in the present studies.

In the present study, we build on past literature to examine a core question that has practical implications: which factors account for the variance between individuals in the way they perceive, tolerate, and communicate risks? We synthesise across the separate factors that have been investigated so far, and present our individual, contextual, cognitive, social framework (ICONS framework, see Fig. 1). We did not make specific hypotheses about the precise direction, or relative influence of these factors in light of mixed past findings. In addition, to our knowledge, no research has integrated the aforementioned factors together (whilst allowing for the potential of bidirectional relationships) in a single study – which was one of the drivers for this research. What we do, however, suggest is that risk perceptions will be a combination of these four factors, and that the most interconnected factor is that of the individual. Individuals vary (demographically, culturally and personally), and these differences influence (1) how an individual appraises a risk; (2) how they integrate contextual information with existing risk perceptions and appraisals; (3) whether and where to seek or share information. Across two pre-registered studies, we investigate risk perceptions both as a static concept (Study 1), but also as a dynamic one, examining the extent to which perceptions are updated in light of new information about harm (Study 2), with the intention of answering the following:

1. Which dimensions underlie risk perceptions for quotidian risks?
2. What is the relative contribution of individual, contextual, cognitive and social factors in shaping risk perceptions?
3. How individuals decide what is an acceptable level of perceived risk (that is, an individual’s *risk tolerance*)?
4. What are the downstream consequences of risk perceptions for risk communication, (i.e. how information is spread), and which factors drive this?

We find that consumer risk perceptions can be explained by three dimensions: benefits, dread and responsibility. The precise nature of these risk perceptions is shaped by a range of individual, cognitive and contextual factors, and show for the first time that these interact with each other. The results of these interactions influence both how much risk we are willing to tolerate, but also our search and communication of risk information. Our results suggest that the contribution of individual differences in shaping risk perceptions has been unfairly discounted in previous work.

## Results

All analyses were run using R^39^ in R Studio, version 4.2.2 (2022-10-31)^40^. A summary of our findings in relation to our research questions can be found in Table 1.

**Table 1:** Research questions and related findings.

Research question	Summary of findings
(1) Which dimensions underlie risk perceptions for quotidian risks?	Three dimensions explain 92.9% variance: benefits, dread and individual responsibility
(2) What is the relative contribution of individual, contextual, cognitive and social factors in shaping risk perceptions?	Study 1: - Benefits and dread were predicted by both individual and contextual (product category) factors, which interacted (more so for benefits) - Individual responsibility was predominantly predicted by individual factors Study 2: - Benefits were mainly predicted by interactions between individual (gender, age, parental status) and contextual factors - Dread was predicted by individual and contextual factors, but these effects were typically independent
(3) How do individuals decide what is an acceptable level of perceived risk (that is, an individual’s *risk tolerance*)?	Study 1: - Risk tolerance was predicted by individual and contextual (product category) factors, which interacted - Risk intensity^a^ was predominantly predicted by individual factors (age, gender, parental status, worldview, personality) Study 2: - Risk tolerance was predicted by individual and contextual (place of purchase, harm information) factors, some of which interacted - Risk intensity was also predicted by a combination of individual and contextual factors
(4) What are the downstream consequences of risk perceptions for risk communication (i.e. how information is spread), and which factors drive this?	Study 1: - High risk perceptions predicted increased likelihood of risk communication with both personal and impersonal sources - Risk tolerance and intensity were also predictive of likelihood of risk communication, though in different directions for different sources - Personal communication was largely predicted by individual characteristics (demographics, personality) whereas impersonal communication was predicted by both individual and contextual (product category) factors Study 2: - High dread predicted increased likelihood of risk communication with both personal and impersonal sources. Low perceived benefits predicted lower communication only with impersonal sources - Personal communication was predicted by individual characteristics (demographics, personality) and contextual (product, harm information) factors. Impersonal communication was largely predicted by individual differences

^a^Risk intensity (perceived dread + benefits) is the complement of risk tolerance, providing an indication of the potency of a risk when all advantages and disadvantages are taken into account.

### Risk perceptions

#### Latent dimensions

In Study 1 (n = 973), each participant rated a random selection of nine consumer products on a series of 11 characteristics (for data preparation, see Supplementary Materials 2). We found that perceived risk ratings of 54 different products (see Supplementary Materials 1) could be explained by three main dimensions (derived from a principal component analysis [PCA] on the aggregated data across individuals, see Supplementary Materials 3), which explained 92.9% of the variance. We termed the first dimension ‘benefits’, which consisted of ‘benefits’ (very great benefits), ‘familiarity’ (extremely familiar), ‘likelihood of use’ (very frequently) and ‘usefulness’ (extremely useful). The second dimension, labelled ‘dread’, consisted of ‘severity’ (extremely severe), likelihood of injury (extremely likely), ‘worry’ (extremely worried) and ‘known to those at risk’ (known precisely to those at risk). The final dimension we termed ‘individual responsibility’, comprising of ‘responsibility for protection’ (totally my responsibility), ‘blame’ (completely the fault of the individual) and ‘control’ (total control).

#### Predictors of risk perceptions

Having identified these three dimensions underlying risk perceptions in Study 1, we performed structural equation modelling (SEM) to predict (a) benefits, dread and responsibility scores derived from the PCA and (b) personal and impersonal source communication scores, using the BRMS package^41^. For full details on the model specification process, see Supplementary Materials 4. Given the diversity of products included, we included four additional overarching product categories to the model, which on the basis of previous literature were hypothesised to influence risk perceptions: product age (‘old’ versus ‘new’), main purpose (‘household good, appliance or healthcare’ versus ‘leisure, recreation or personal care’), power/fuel (‘involves power/fuel’ versus ‘does not involve power/fuel’) and vulnerable groups (‘vulnerable groups use/interact with more than other groups’ versus ‘vulnerable groups do not use/interact with more’). The categorisation of each product was validated in a separate study (Study 1B, see Supplementary Materials 5). As an example, fridge/freezers were categorised as old, household goods, involving power/fuel and not used/interacted with more by vulnerable groups. In contrast, neodymium magnets in construction toys were categorised as new, leisure goods, non-powered and used/interacted with more by vulnerable groups. UMAP analysis^42^ of how these products clustered can be found in Supplementary Materials 6.

**Figure 2:**
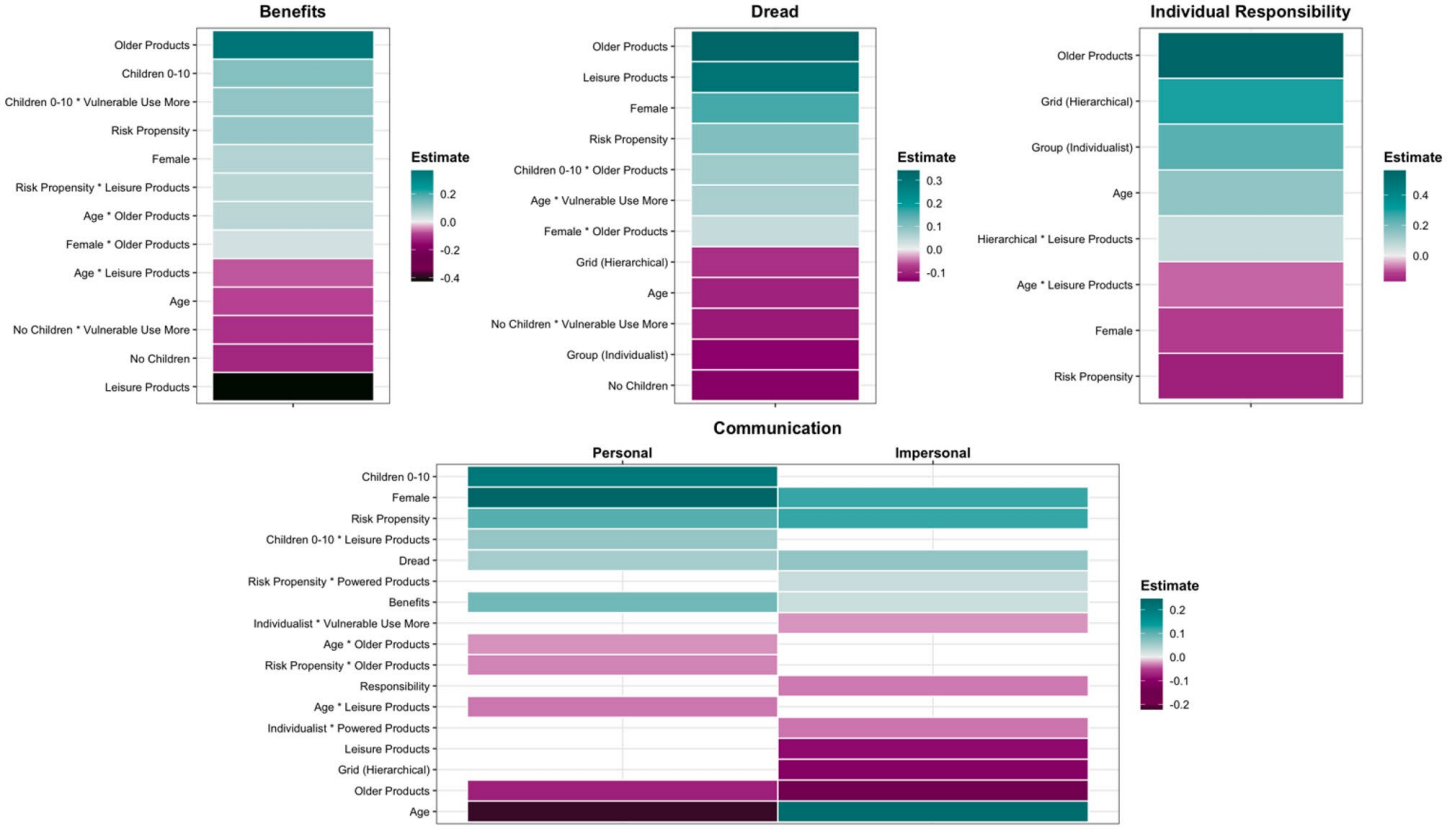
Clear (≠ 0) Predictors of risk perceptions and communication – Study 1. The final model specified was: benefits, dread, responsibility, communication – personal/impersonal ~ (grid worldview + group worldview + risk propensity + age + gender + children) * (product age + main purpose + power/fuel + vulnerable groups) + (1 + product age + main purpose + power/fuel + vulnerable groups|ID) + (1|Product). Positive (negative) estimates indicate high (low) benefits, high (low) dread, high (low) individual responsibility and high (low) likelihood of communicating risk information (both seeking and sharing).

Higher benefits and dread were perceived by those who were younger, females, parents or those high in risk propensity (for an overview, see Fig. 2). Dread was additionally influenced by cultural worldview, with hierarchical and individualist worldviews associated with lower dread. The level of benefits perceived for certain types of products (older, leisure, or those used more by vulnerable groups) was less consistent and differed according to age, gender, parental status and risk propensity. In contrast, levels of dread were consistently predicted by product type, with older or leisure products more dreaded than newer or household products.

Individual characteristics were most influential for perceptions of perceived individual responsibility. Higher levels of individual responsibility were perceived by those who were older, male, had an individualist or hierarchical worldview or those low in risk propensity. There was less of an effect of product category, though older products were perceived as more of the responsibility of individuals versus newer products.

To summarise, differences in perceived individual responsibility were primarily driven by individual differences. In contrast, differences in perceived dread and benefits were predicted by a combination of individual differences and product category. The fact that high benefits were associated with high levels of dread could seem counter-intuitive, but can be reconciled with reference to risk tolerance, recognising that there is an active trade off between positive and negative aspects of risk.

Risk perceptions in Study 1 were measured in the absence of any contextual information – participants were given only the name of a product and a brief description, before rating them. In reality, product risk is assessed within a far richer context, where information from the environment and others around us is integrated to form an overall perception of a particular product. Study 2 therefore included more contextual information at Time 1 (product purchase location: online versus bricks and mortar store), with information about a harm incurred introduced at a second time point. This harm information related to *cause* (arising from product non-compliance versus the user’s actions), and *who experienced the harm* (friend [known] versus unknown other).

We therefore created composite benefit and dread scores for each time point (Time 1 – initial perception, Time 2 – perception post-harm information). We ran a Bayesian multivariate mixed effect model to predict benefits and dread, using the BRMS package^41^. For full model specification details, see Supplementary Materials 4. As expected, there was a strong effect of time, with higher dread and lower benefits at Time 2.

Higher overall perceived benefits (i.e. across time points) were perceived by those high in risk propensity, though the majority of the effects of individual differences occurred in relation to time. Older adults, females or those with children under 10 perceived fewer benefits at Time 2. There were consistent interactions between the contextual factors (place of purchase, cause of harm, who experienced harm) and time, with reduced benefits at Time 2 when the product was bought online, harm caused by non-compliance, or a friend experienced the harm.

**Figure 3:**
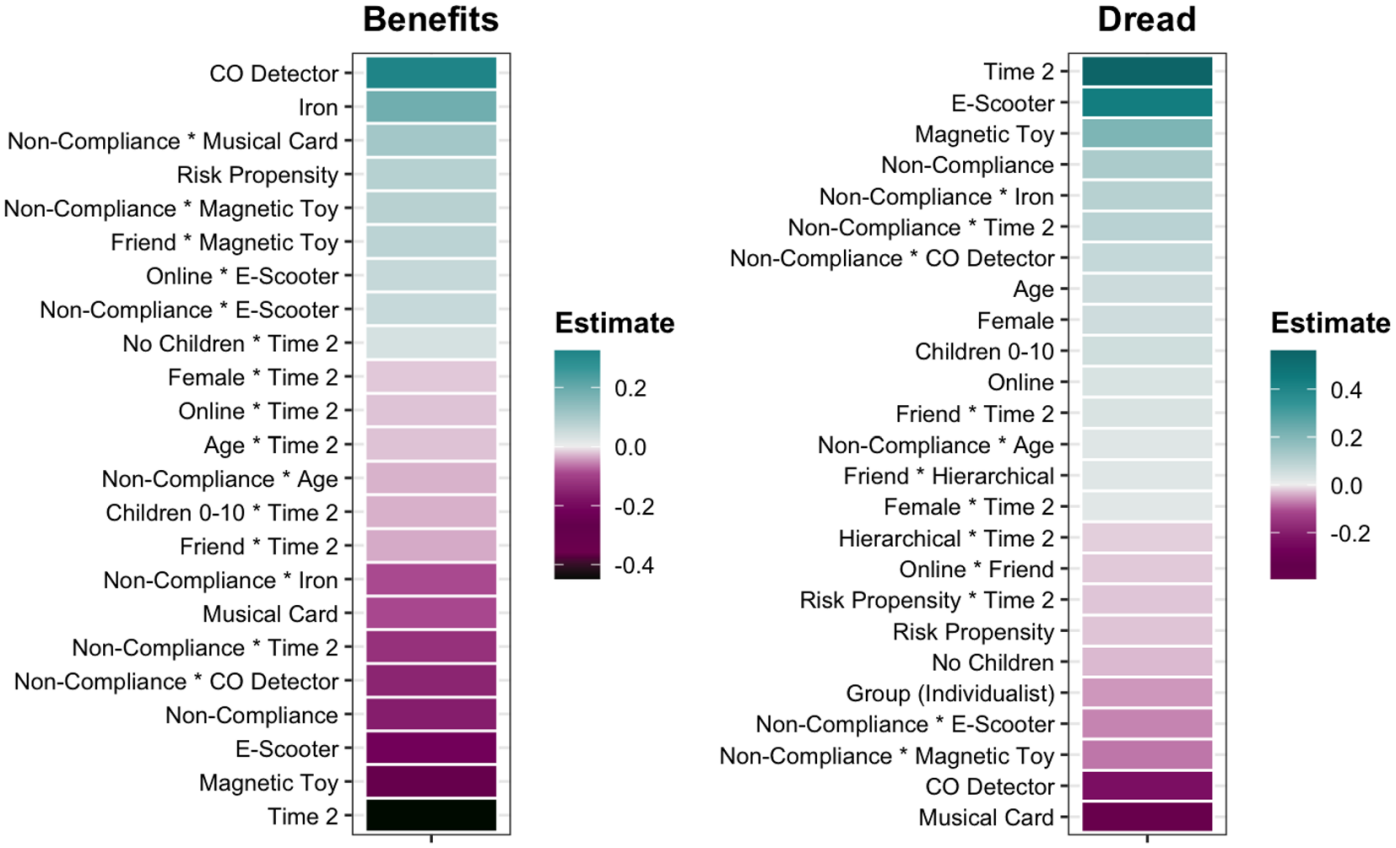
Clear (≠ 0) predictors of overall risk perceptions – Study 2. Estimates were derived from the model: dread, benefits ~ ((product + place of purchase + harm cause + harm experience)^2) + time + (time * harm cause) + (time * harm experience) + (place of purchase * harm cause * time) + (place of purchase * harm experience * time) + (age + gender + children + risk propensity + grid worldview + group worldview) * (place of purchase + harm cause + harm experience + time) + (1|ID). Positive estimates indicate higher dread and benefits.

Higher overall dread levels were perceived by those who were older, females, parents or those low in risk propensity and for those products bought online (for an overview, see Fig. 3). Again, as expected, the contextual factors interacted with time, with higher dread levels at Time 2 when harm was caused by non-compliance and if a friend experienced the harm. Additionally, the specific product also influenced dread levels, though the direction of this effect varied by non-compliance.

Consistent with previous literature using the psychometric paradigm, our findings highlight that risk perceptions are influenced by a variety of qualitative factors, which are not directly related to objective measures of risk (e.g. probability of occurrence). These qualitative factors include both contextual factors (product category membership, product, presence of harm information), as well as individual factors, such as those relating to demographics, personality and cultural worldview. Our results emphasise the need to consider not just differences between risks, but also differences amongst individuals when investigating risk perceptions. Moreover, for the first time we show that these factors interact, supportive of our integrative approach.

### Risk tolerance and intensity

Whether an individual decides to take an action or not based on how they consider the risk is only partly a function of perceived dread. Other practical factors will play a role, such the opportunities (utility) gained. What has often been overlooked in the psychological risk literature is how individuals decide what is an acceptable level of perceived risk, that is, an individual’s *risk tolerance*^14^. This is conceptualised as the trade-off between perceived benefits (utility) and perceived dread (cost) when individuals decide whether to use a product, even if they perceive a risk^5^. We investigated this in exploratory analyses: in Study 1, risk tolerance was operationalised as the benefits dimension score [B] minus the dread dimension [D] score. In Study 2, the benefits dimension comprised of ‘benefits + likelihood of use’ and the dread dimension comprised of ‘worry + severity + hazardousness’, with tolerance again calculated as benefits minus dread. In both cases, high values indicate high risk tolerance (high levels of perceived dread, but still high benefits). As a complement, combining both perceived dread and benefit dimensions gives a general indication of the potency of risk of a product, taking all advantages and disadvantages into account – what we refer to as ‘perceived risk intensity’. In this way, when making a purchasing decision, risk intensity is the formation of the overall impression of the product’s risk, whereas risk tolerance is the calculation to pursue the risk given the opportunities afforded by it.

Rather than being mere statistical constructs, we argue that both risk tolerance and risk intensity help further elucidate the dynamic, multi-dimensional nature of how individuals perceive risk and provide a more nuanced view. Risk tolerance captures the degree to which perceived benefits are powerful enough to outweigh risks of hazards. Risk intensity captures the intensity of response, irrespective of valence – whether positive (low levels of dread and high levels of benefits) or negative (high levels of dread and low levels of benefits). To illustrate an example from our dataset, irons are perceived fairly equally for dread and benefits, as are musical greeting cards. The risk tolerance associated with these two products is thus fairly similar. However, the risk intensity of irons (which are perceived as high dread and high benefits) is much higher than for musical greeting cards.

For Study 1, we conducted structural equation modelling to predict (a) risk tolerance, perceived risk intensity and responsibility scores and (b) personal and impersonal communication scores using the BRMS package^41^. For full model specification details, see Supplementary Materials 4.

**Figure 4:**
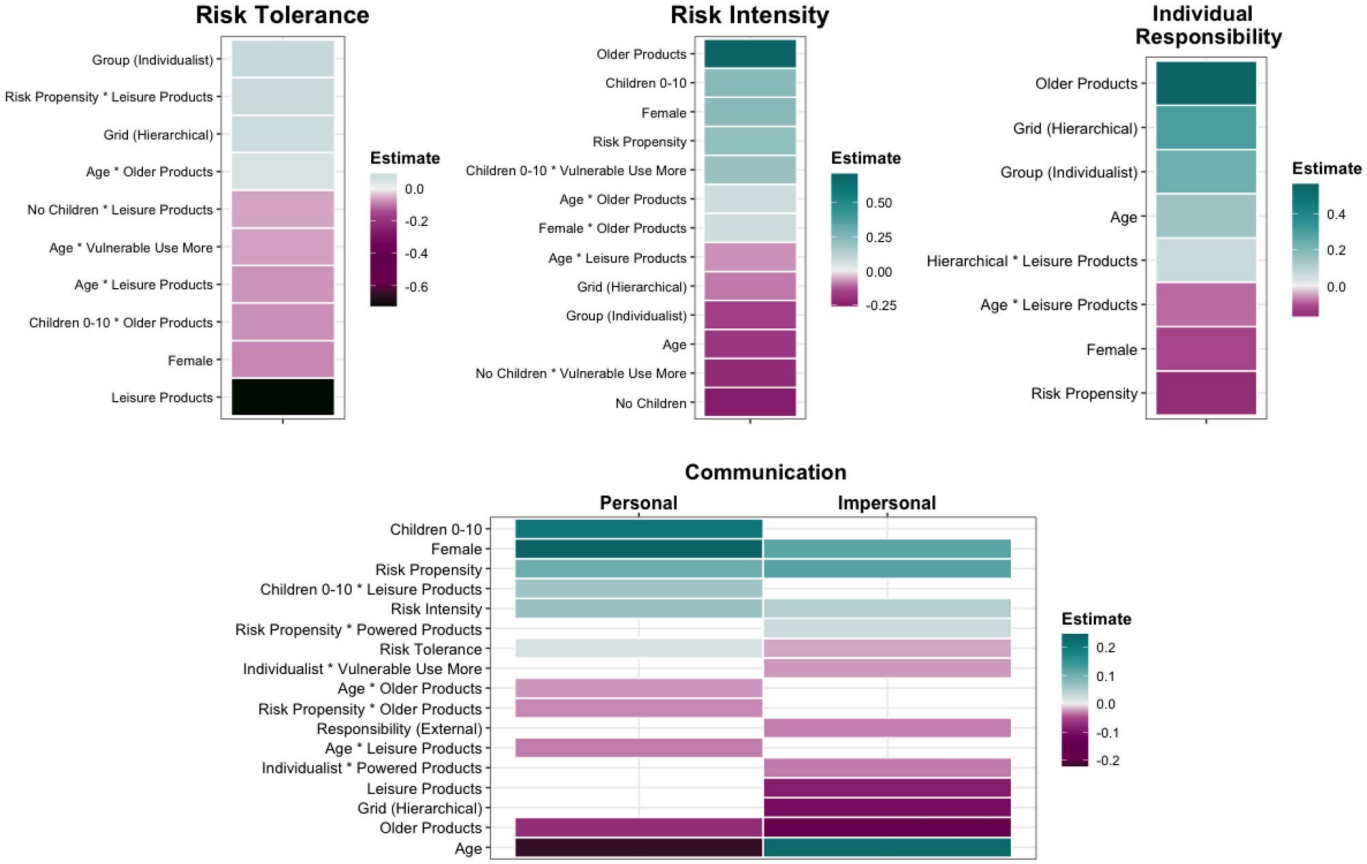
Clear (≠ 0) Predictors of risk tolerance, intensity, responsibility and communication – Study 1. The final model specified was: risk tolerance, risk intensity, responsibility, communication – personal/impersonal ~ (grid worldview + group worldview + risk propensity + age + gender + children) * (product age + main purpose + power/fuel + vulnerable groups) + (1 + product age + main purpose + power/fuel + vulnerable groups|ID) + (1|product). Positive estimates indicate increased risk tolerance and intensity, increased individual responsibility and increased likelihood of communicating risk information (both seeking and sharing).

Whilst risk tolerance was predicted by individual differences (demographics, cultural worldview and personality), this was most frequently as part of interactions with product category membership (Fig. 4). For instance, the older an individual was, the lower their perceived tolerance for the risk posed by newer or leisure products, or those used by vulnerable groups.

Risk intensity was primarily predicted by individual differences, with higher risk intensity perceived by those who were young, females, parents of children aged 0–10, those with an egalitarian or communitarian worldview, or those high in risk propensity (for an overview, see Fig. 4). There were higher levels of risk intensity for older products. Whether vulnerable groups interacted with or used the product more was also predictive of risk intensity, but only in relation to parental status, whereby parents of children aged 0–10 perceived higher intensity for products used more by vulnerable groups.

Differences in perceptions of individual responsibility were extremely similar to those reported for the original SEM model in the previous section – with individual characteristics far more influential compared to product category characteristics.

In Study 2, risk tolerance and perceived risk intensity was captured at two time points (both pre and post presentation of the harm information). Using the BRMS package^41^, we used multivariate modelling to predict risk tolerance and intensity scores, assessing the effect of presentation of the harm information by including interactions with time within this model. For full model specification details, see Supplementary Materials 4.

**Figure 5:**
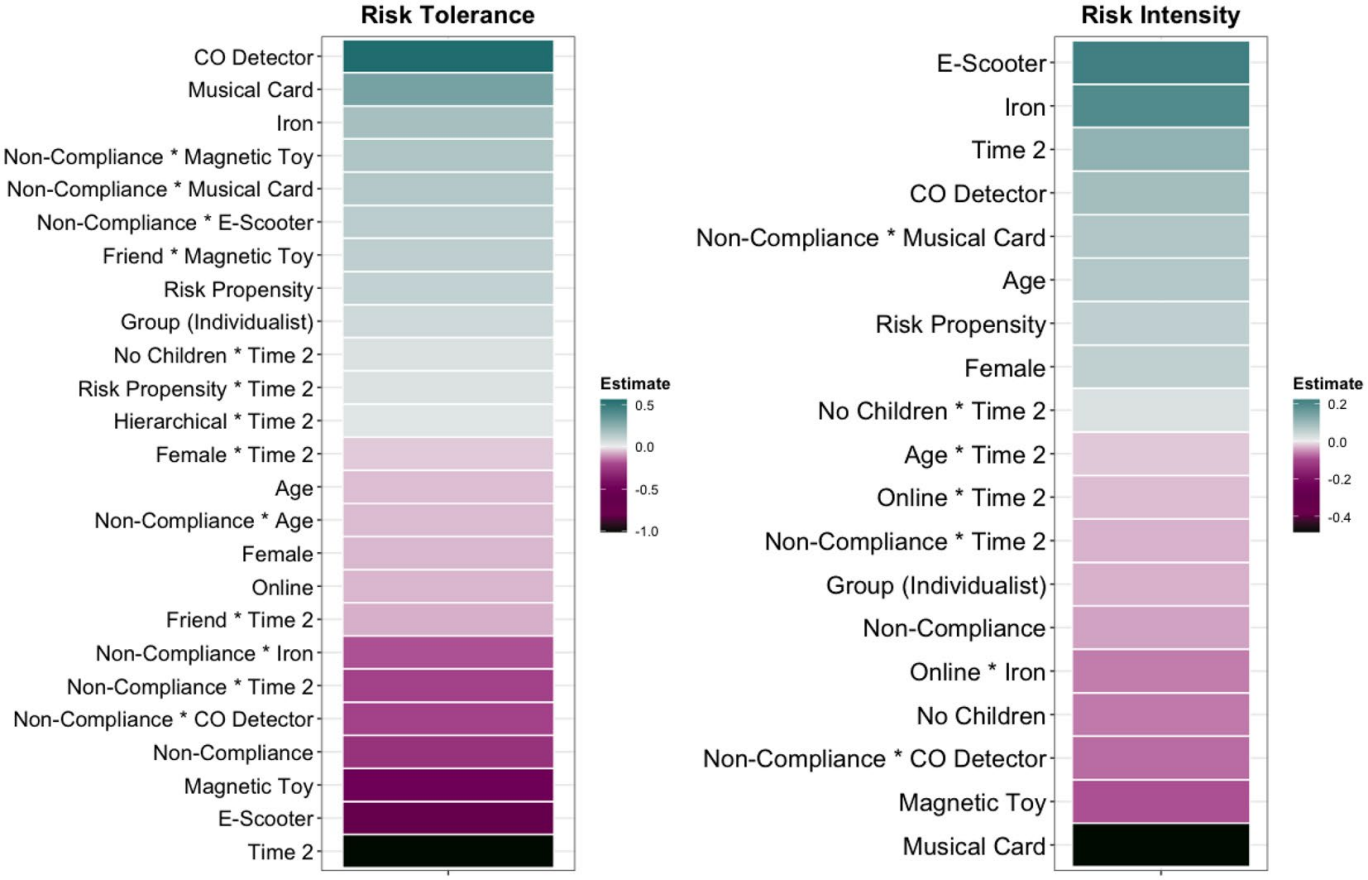
Clear (≠ 0) predictors of risk tolerance and perceived risk intensity – Study 2. Estimates were derived from the final model specification: risk tolerance, risk intensity ~ ((product + place of purchase + harm cause + harm experience)^2) + time + (time * harm cause) + (time * harm experience) + (place of purchase * harm cause * time) + (place of purchase * harm experience * time) + (age + gender + children + risk propensity + grid worldview + group worldview) * (place of purchase + harm cause + harm experience + time) + (1|ID). Positive numbers refer to higher risk tolerance and perceived risk intensity.

As expected, risk tolerance was lower at Time 2, with both individual and contextual factors consistently predictive of tolerance. At Time 2, males, non-parents, those with an hierarchical worldview, or those high in risk propensity displayed higher risk tolerance. Similarly, clear effects of contextual factors were also observed. Overall, products purchased online showed lower risk tolerance. At Time 2, lower risk tolerance was observed for non-compliant products or when a friend experienced the harm (see Fig. 5).

As envisaged, perceived risk intensity was also higher at Time 2. Similar to risk tolerance, both individual (demographic, personality) and contextual factors predicted perceived risk intensity. Females perceived higher levels of risk intensity overall. At Time 2, older adults showed reduced risk intensity (see Fig. 5). In terms of contextual factors, individuals perceived lower levels of risk intensity at Time 2 for products purchased online and those which turned out to be non-compliant.

### Likelihood of risk communication and attributions of responsibility

In Study 1, differences in likelihood of seeking and sharing of product risk information to *personal* sources were primarily driven by individual differences (Fig. 4). In terms of demographics, females, younger adults and parents of children under 10 were more likely to seek information from, and share it with personal sources versus males, older adults or non-parents. When it came to *impersonal* sources, product category membership played more of a role, with lower likelihood of communication for older, leisure products.

High levels of perceived dread/benefits were associated with increased likelihood of risk communication with both personal and impersonal sources. Relatedly, risk tolerance and intensity were also predictive of likelihood of seeking and sharing of product risk information; higher perceived risk intensity was associated with higher likelihood of communication with both sources, whereas higher risk tolerance was associated with higher likelihood to personal sources, and lower likelihood of communication with impersonal sources.

In Study 2, we measured the communication of risk only after presentation of the harm information. Using the BRMS package^41^, we performed SEM to predict (a) dread and benefit scores at Time 2, (b) personal and impersonal communication scores and (c) responsibility attributions to external parties and the user. In light of Fig. 3, for clarity, we present the results of (b) and (c), with the full version available in Supplementary Materials 7.

**Figure 6:**
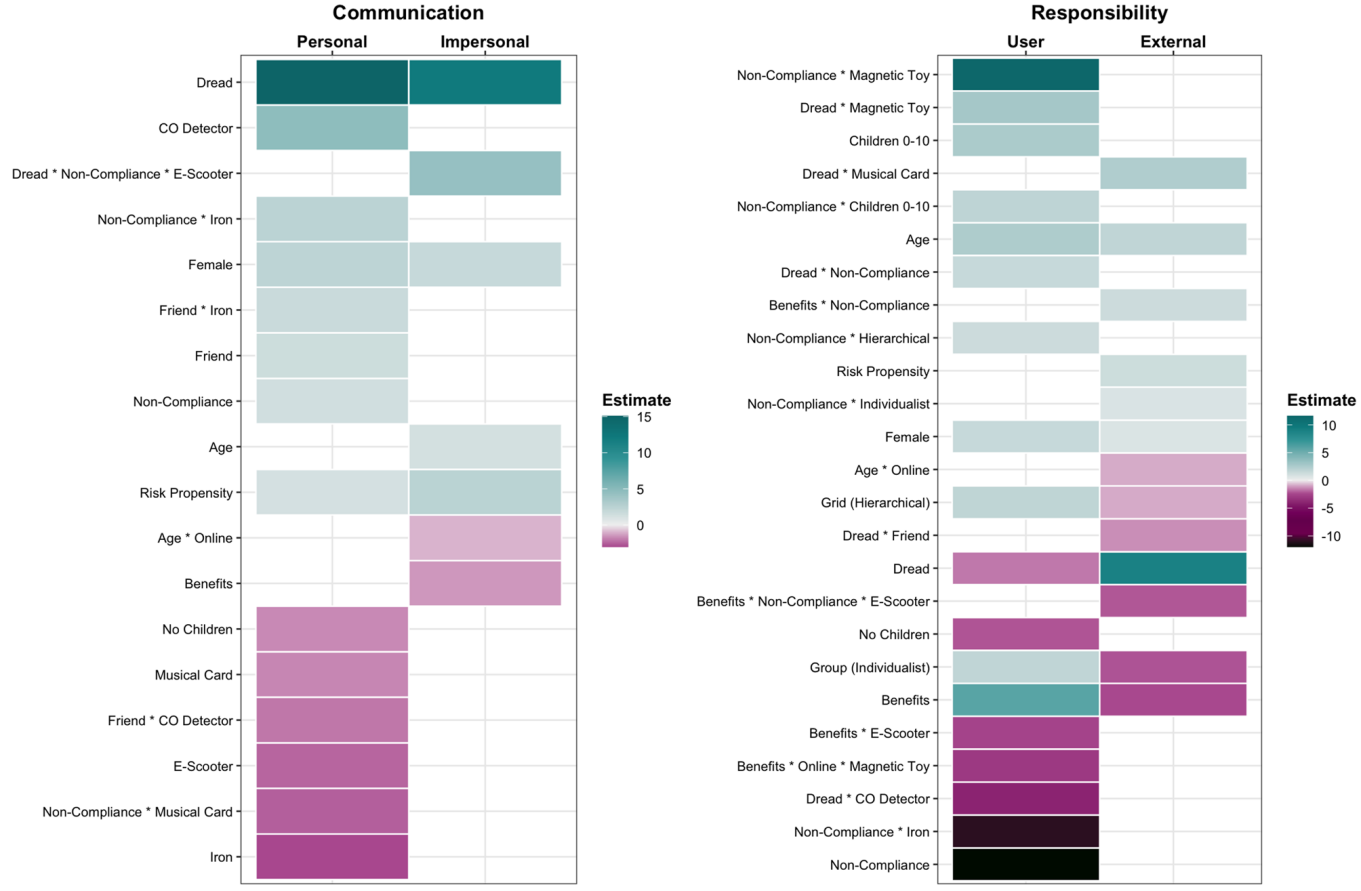
Clear (≠ 0) predictors of communication and responsibility attributions – Study 2. Estimates were derived from the final models: communication – personal/impersonal, responsibility – user/external ~ ((product + place of purchase + harm cause + harm experience)^2) + (dread + benefits) + (age + gender + children + risk propensity + grid worldview + group worldview) * (place of purchase + harm cause + harm experience)). Higher estimates indicate increased likelihood of communicating risk information (both seeking and sharing) and increased responsibility.

In Study 2, differences in risk communication with *personal* sources were partially driven by individual differences, with females, parents of children under 10 or those higher in risk propensity more likely to seek information from, and share it with personal sources (see Fig. 6). However, also influential were product and contextual differences (cause of harm, who experienced the harm), though the direction of these effects depended on the specific product in question. For communication with *impersonal* sources, individual differences were most influential, with higher likelihood of communication for females, older adults and those higher in risk propensity.

High dread levels predicted higher likelihood of both personal and impersonal risk communication, though benefits predicted engagement only with impersonal sources, with high benefits associated with a lower likelihood of risk communication.

Both dread and benefits predicted attributions of responsibility, with higher dread predicting higher external responsibility and higher benefits predictive higher user responsibility. Individual differences (demographics, cultural worldview) consistently predicted attributions of responsibility, such that being female, older, a parent or having an individualist/hierarchical worldview were predictive of higher levels of user responsibility. Contextual factors were also important: product and cause of harm consistently predicted attributions of responsibility. When the harm was caused by product non-compliance, generally external responsibility attributions were higher, though there were interactions by product.

## Discussion

Across two studies, we find that risk perceptions for consumer products are multi-faceted and are influenced by a combination of cognitive appraisals, individual differences, social and contextual factors. Our study examines a far broader range of predictors compared to previous research, which has typically only focused on one of these factors at a time. The fragmented nature of research and lack of integrated analysis in the risk community has long since been noted^37^; the development of ICONS provides a framework in which we demonstrate that these factors have interactive effects over and above the singular effects documented in previous literature. Broadly, risk perceptions for consumer products comprise of three components: benefits, dread, and individual responsibility (Study 1). These perceptions are not static but rather change in response to new information about harm (Study 2), though the extent to which they do differs according to cognitive appraisals and individual characteristics (demographics, cultural worldview and risk propensity).

Replicating prior results from the cognitive theory of risk, we find that risk characteristics (e.g. severity of harm, familiarity, worry) account for a considerable proportion (93%) of variance in risk perceptions. However, we also find that individual characteristics play an important role in predicting risk perceptions, supportive of our integrative ICONS framework. Personality (risk propensity) and socio-demographic characteristics, such as age, gender and whether one is a parent of a child under 10 consistently predicted the three components. Whilst the influence of age and gender has been noted in the literature on risk perceptions^17–19^, up until now the effect of parental status has been generally overlooked. Our findings are however in line with results from the health literature, which show that parents make more risk averse decisions for their children than for themselves^43^ and indeed have significantly higher risk perceptions, for instance of Covid-19^44^. We also observe that individual’s cultural worldview shapes risk perceptions^45–47^, predominantly for the individual responsibility component.

Whilst the concept of ‘risk tolerance’ is certainly not new, particularly in the finance domain^48^, prior psychological literature has almost wholly focused on risk perceptions, at the expense of risk tolerance. Part of this focus may be a result of the lack of consistency in how the term has been defined or used previously; a problem observed across many disciplines. Yet a fuller understanding of public attitudes towards risk cannot be achieved without knowing how risk perceptions are integrated by the individual in order to calculate an ‘acceptable (tolerable) level of risk’. This requires capturing not just whether the positive aspects of risk outweigh the negatives (risk tolerance), but also the strength of these two aspects (risk intensity). Although we find evidence that risk tolerance reflects dispositional (individual) elements^49^, such as age, gender and parental status, these effects were frequently moderated by situational elements^50^ such as product category membership and harm information. In contrast, risk tolerance’s complement ‘risk intensity’ reflected more individual characteristics. Although not tested in this research, we would also expect other contextual factors such as social trust to influence an individual’s perceived risk tolerance, given that social trust has previously been strongly correlated with risk perceptions^51–53^. Of particular note is the fact that both risk tolerance and risk intensity predicted likelihood of risk communication. Given that in the absence of in-depth knowledge or direct experience, we are reliant on mediated information from others^24,54^, this has the potential to lead to further risk dissemination and thus potentially risk amplification^55^.

We specifically sought to use a domain general measure of risk propensity^20^ given domain specific measures did not exist for product safety, and existing measures (e.g. those developed for financial or health domains) were not neatly applicable. Our findings regarding risk propensity were seemingly inconsistent – higher levels of risk propensity were predictive of higher dread (Study 1) and high likelihood of seeking information and sharing risk information with personal and impersonal sources (Study 1), yet also predicted higher levels of risk tolerance post harm information (Study 2). It is often assumed that a willingness to take risks (high risk propensity) necessarily entails low levels of perceived risk, though our results indicate that the two are independent. One possibility is that rather than capturing the *likelihood* of taking risks, the risk propensity measure captures *experience* of risks, whereby a high risk propensity means that only very extreme risks are perceived as risky. In the current research, the risks highlighted were far from extreme, limiting the relevance of the measure. Another possibility is that those higher in risk propensity are simply more accepting of risks, as supported by the predictive relationship seen for risk tolerance shown in Study 2.

The role of social processes in the formation and maintenance of risk perceptions has previously been considered within the literature, but typically examining only perceived risk as a predictor, and for one or two particular sources. For instance, greater severity or perceived risk is linked with increased information seeking^56,57^, a finding most recently exemplified during the Covid-19 pandemic, where greater risk perceptions were associated with increased information search across social media, broadcast media and other individuals (e.g.^58^). Here, we extended this research to examine how individual characteristics might also contribute to which sources individuals are likely to seek information from and communicate risk to, (independent of information insufficiency and risk perceptions, thus considering a more direct relationship than in the risk information seeking and processing [RISP] model^57^. Crucially, both demographics (e.g. age, gender, parental status) and risk propensity were consistent predictors of likelihood of these social interactions, which has implications for how expert sources disseminate information about risk. The implications of these findings are first that, given increased homophily in social networks, differences in risk perception between groups of people may be amplified. And second, rather than taking a one size fits all approach to risk communication, our results indicate that such communications will be more effective when they are tailored to certain groups of individuals (for instance males/younger generations), given they show reduced intentions to seek and/or share risk information.

We investigated consumer risk perceptions solely in participants from the United Kingdom using the online platform Prolific Academic^59^. Whilst in the first study, the sample was nationally representative in terms of age, gender and ethnicity, participation online generally means that participants are from relatively highly educated, higher socio-economic status groups. As a result, our study might be argued to predominantly reflect individual experiences of those from WEIRD (Western, educated, industrialised, rich and democratic) societies^60^. This should be born in mind when interpreting the results, especially those concerning the influence of cultural worldview, given that by definition WEIRD societies reflect a particular type of culture. Indeed, the cultural cognition thesis has been criticised for primarily focusing on ‘American’ culture^61^, neglecting to consider cross-cultural differences.

Whilst we believe that the current work moves us closer towards a more comprehensive understanding of risk perceptions, we do not claim to have explained the full complexities of risk perceptions with the ICONS framework. There was a limit to how much we could measure the richness of differences between individuals, and thus we focused on factors identified from previous risk theories as potentially influential. In addition, by focusing on individuals, our current data cannot speak to how the factors which shape risk perceptions will unfold at a wider, population level. Our future work using agent-based modelling seeks to investigate how individual level processes give rise to population level effects, enabling us to test theories such as the social amplification of risk^36^.

Nevertheless, we see this study as the start of an ambitious, more holistic approach to risk, which can be built upon. For too long, risk research has tended to narrow its focus and not made the most of the wealth of findings derived from different approaches, nor the additional insights that can be gained from considering the functionality of risk (i.e. in terms of risk tolerance). We firmly believe integrating these perspectives will greatly advance the field, and pay dividends to the development of more effective risk management and communication strategies.

## Methods

### Participants

In Study 1, a nationally representative United Kingdom sample (on the basis of age, gender and ethnicity) of 1005 participants was recruited from the online participant platform Prolific Academic (www.prolific.ac) and in Study 2, a sample of 3360 participants (balanced across sex) were recruited from Prolific Academic. For both studies, participants were required to be over 18, in order to meet ethical requirements. Participants were paid commensurate with time taken to complete the study, £3.25 – approximately £8 per hour (Study 1), £0.80 – £7 per hour (Study 2). Participants were excluded for completing the studies unreasonably quickly (< 12 min [Study 1], < 3.5 min [Study 2]) or if their responses for any of the products had a SD < 0.5 (Study 1). For final sample characteristics, see Table 2. Informed consent was obtained from all participants. Ethical approval was granted from Department of Psychology (Royal Holloway, University of London) for all studies within the manuscript. All methods were carried out in accordance with relevant guidelines and regulations.

**Table 2:** Characteristics of sample for Studies 1 and 2.

Demographic		National representative %	Percentage	
			Study 1	Study 2
	Total sample post exclusions (n)	N/A	973	3255
Gender	Male	49.2	48.2	49.5
	Female	50.8	51.0	49.7
	Other	N/A	0.6	0.4
	Prefer not to say	N/A	0.2	0.2
Age	Under 18	21.4	n/a	n/a
	18–24	9.4	12.9	11.7
	25–34	13.5	17.2	26.8
	35–44	14.0	18.6	23.5
	45–54	13.7	16.2	18.2
	55–64	11.6	24.6	14.0
	65 +	16.3	10.4	5.3
	Prefer not to say	N/A	0.2	0.1
Ethnicity	White	85.4	84.1	88.4
	Mixed/multiple ethnic groups	2.3	2.7	2.7
	Asian/Asian British	7.8	7.7	4.7
	Black/African/Caribbean/Black British	3.5	3.5	3.0
	Other ethnic group	1.0	1.2	0.3
	Prefer not to say	N/A	0.8	0.6
Children under the age of 18	Yes – aged between 0–10	N/A	15.8	17.5
	Yes – aged between 11–18		10.2	10.7
	Yes – aged between 0–10 and 11–18		N/A	3.8
	No		73.5	66.8
	Prefer not to say/missing/incompatible options selected		0.5	0.9

### Stimuli

#### Study 1

We selected fifty-four products from a combination of previous literature and those identified by the Office for Product Safety and Standards [OPSS] as of particular interest for research. OPSS is the UK’s national regulator for product safety and standards, which forms part of the Department of Business and Trade. These products were of interest to OPSS for a range of reasons, including: topicality (e.g. relating to COVID-19 – e.g. UV light sanitisers), a lack of existing knowledge/understanding (e.g. electric scooters), frequency of injury relating to product (e.g. button batteries), electrical safety (e.g. USB chargers) and potential for risk from misuse (e.g. balcony barbeques). Each product was presented with a short descriptive sentence to provide context, though no mention of associated risks or benefits was made. These sentences were developed using the Delphi method (OPSS regulators and experts) and in reference to existing research. For the full list of products and associated descriptions, see Supplementary Materials 1.

#### Study 2

Six products (see Table 3) were selected according to (a) their scores on the three components identified in Study 1, equivalent to one high and one low scoring product for each component (b) their main purpose categorisation identified in Study 1B, equivalent to three leisure products and three household products. These products were presented either in the context of buying online or on the high street. Product harm information was presented as either being caused by: non-compliance, or the user, and happening to: an unknown other, or a friend (see Table 3). All conditions were manipulated between participants.

**Table 3:** Products, associated descriptions and harm – Study 2.

Product	**Experience—unknown other** (You decide to purchase the product. However, having purchased the PRODUCT, you subsequently see a media story about a consumer …)		**Experience—friend** (You decide to purchase the product. However, having purchased the PRODUCT…)	
	Non-compliance	User related	Non-compliance	User related
**TOUR-X electric scooter (high dread).** A device consisting of two or three wheels, handlebars and a floorboard which is stood on while riding, powered by an electric motor. Typically used for personal transport	…who owns the same scooter but crashed on it, and suffered injuries after being unable to stop due to high speed.		… your friend tells you that they own the same scooter but crashed on it, and suffered injuries after being unable to stop due to high speed.	
	This story reports that the product does not meet the requirements of the Supply of Machinery Regulations 2008. The scooter goes over the maximum speed requirement due to the speed not being limited.	This story reports that the rider was exceeding the maximum speed limit.	Your friend subsequently found out that the product does not meet the requirements of the Supply of Machinery Regulations 2008. The scooter goes over the maximum speed requirement due to the speed not being limited.	Your friend tells you that they were exceeding the maximum speed limit.
**GREETIZE musical greetings card (low dread).** A greetings card which plays music when it is opened. Such cards typically contain a small device embedded in the card, powered by one or more small button batteries	…who bought the same musical greetings card, and their child placed the button cell batteries in their mouth and swallowed, causing damage to the gastrointestinal tract.		…you find out that a friend bought the same musical greetings card, and their child placed the button cell batteries in their mouth and swallowed, causing damage to the gastrointestinal tract.	
	This story reports that the product does not meet the requirements of the Toys (Safety) Regulations 2011. According to regulations, the product makes the batteries too easy for children to access from their compartment.	This story reports that it is possible for children to be able to access the batteries from their compartment.	Your friend subsequently found out that the product does not meet the requirements of the Toys (Safety) Regulations 2011. According to regulations, the product makes the batteries too easy for children to access from their compartment.	Your friend tells you that it is possible for children to be able to access the batteries from their compartment.
**PLUG’D USB wall charging plug and cable (high benefits).** USB wall charging plug and cable, purchased separately from the device they are intended to power/charge	…Who owns the same product, and suffered burns from a fire started by the wall charging plug.		…Your friend tells you that they also own the same product, and suffered burns from a fire started by the wall charging plug.	
	This story reports that the product does not meet the requirements of the Plugs and Sockets etc. (Safety) Regulations 1994 – the plug does not incorporate a suitable protective fuse.	This story reports that the plug socket was overloaded and the charger was left plugged in for a long period of time.	Your friend subsequently found out that the product does not meet the requirements of the Plugs and Sockets etc. (Safety) Regulations 1994 – the plug does not incorporate a suitable protective fuse.	Your friend tells you that the plug socket was overloaded and the charger was left plugged in for a long period of time.
**MAGNE-BUILD magnetic construction toy (low benefits).** A toy made from neodymium magnets which are small, super strong, spherical magnets. These magnets can be separated and put together into various shapes and patterns	…who owns the same construction toy. Their young child placed the magnets in their mouth and swallowed, causing internal injuries.		You find out that a friend owns the same construction toy. Their child placed the magnets in their mouth and swallowed, causing internal injuries.	
	This story reports that the product does not meet the requirements of the Toys (Safety) Regulations 2011. The magnetic flux (strength of the magnet) is far greater than the legal maximum.	This story reports that the child’s internal injuries were caused by the magnets being drawn together in their digestive system.	Your friend subsequently found out the product does not meet the requirements of the Toys (Safety) Regulations 2011. The magnetic flux (strength of the magnet) is far greater than the legal maximum.	Your friend tells you that their child’s internal injuries were caused by the magnets being drawn together in their digestive system.
**THERMA-STEAM—Electric iron (high individual responsibility).** An electrical appliance, which uses heat to press folds out of clothes	…Who owns the same product and suffered second-degree burns from the iron.		…Your friend tells you that they also own the same product and suffered second-degree burns from the iron.	
	This story reports that this product does not comply with the relevant European Standard EN 60335. The plastic part of the iron can overheat.	This story reports that the burns were caused by mishandling of the iron.	Your friend subsequently found out that the product does not comply with the relevant European Standard EN 60,335. The plastic part of the iron can overheat.	Your friend tells you that the burns were caused by mishandling of the iron.
**VULCAN—Carbon monoxide detector (low individual responsibility).** A device which monitors and measures levels of carbon monoxide in the air, sounding an alarm if it detects the presence of carbon monoxide	…Who owns the same product. They were inadvertently exposed to carbon monoxide for an excessive amount of time, and suffered carbon monoxide poisoning.		…Your friend tells you that they also own the same product. They were inadvertently exposed to carbon monoxide for an excessive amount of time, and suffered carbon monoxide poisoning.	
	This story reports that the product does not comply with the European Standard EN50291. The carbon monoxide detector does not give an alarm promptly enough when exposed to low concentrations of carbon monoxide.	This story reports that the detector’s batteries were low, such that it did not give an alarm promptly enough when exposed to low concentrations of carbon monoxide.	Your friend subsequently found out that the product does not comply with the European Standard EN50291. The carbon monoxide detector does not give an alarm promptly enough when exposed to low concentrations of carbon monoxide.	Your friend tells you that the detector’s batteries were low, such that it did not give an alarm promptly enough when exposed to low concentrations of carbon monoxide.

### Procedure

Both studies were run using the Qualtrics online survey platform (www.qualtrics.com). Before beginning the main study, participants were asked a series of demographic questions. They were asked to indicate: gender (male/female/other – please specify/prefer not to say); age, ethnicity (White, Mixed, Asian, Black and Other/Prefer not to say) and if they have children under the age of 18 (Yes – aged between 0 and 10/Yes – aged between 11 and 18/No/Prefer not to say). They were also asked for their Prolific ID, used for payment and then completed a captcha question.

#### Study 1

Participants were randomly presented with nine of the fifty-four products to rate. Each product was rated on a series of 11 risk characteristics, using a 7-point Likert scale (as used previously in Refs.^58,59^. Risk characteristics were primarily selected on the basis of their use in previous psychometric studies of consumer products^62–66^. The complete list of characteristics and response scales can be found in Table 4, with a comparable number of characteristics to those in Wogalter et al.^62^ and a similar length questionnaire to that used by Feng et al.^67^. To reduce the likelihood of participants simply clicking through without reading the questions, the next page button only appeared after 25 s.

**Table 4:** Product characteristics – Study 1.

Characteristics
Benefits How great are the benefits associated with the above product to you personally? *(No benefits at all, to Very great benefits)*
Severity How *severely* (i.e. degree, extent or magnitude) might you, or anyone else, be injured by the above product? *(Not at all severe to extremely severe)*
Familiarity How *familiar* are you with the above product? *(Not at all familiar to extremely familiar)*
Known to those at risk To what extent are the risks associated with the above product known precisely to the persons who are exposed to the risk? *(Completely unknown to known precisely)*
Control If exposed to the product, to what extent can you, by personal skill, diligence or training, avoid the hazards associated with the above product? That is, how much control do you have over being injured by the above product? (*No control at all to total control)*
Likelihood of injury How *likely* are you or anyone else to receive *any* injury from the above product, including all *minor* ones (requiring little or no first aid) and *major* ones (requiring emergency treatment)? *(Never—Extremely likely)*
Worry How worried are you about potential risks associated with use of the above product? *(Not worried at all, to Extremely worried)*
Blame To what extent would an injury associated with the above product be the fault of the individual or the fault of external parties, such as the retailer, manufacturer or government regulator? *(Completely the fault of the individual – Completely the fault of external parties)*
Responsibility for protection To what extent is it your responsibility, or the responsibility of others (such as the retailer, manufacturer or government regulator), to protect you from harm associated with the above product? *(Totally my responsibility to Totally the responsibility of external parties)*
Likelihood of use If you own (or were to own) the above product, how *often* would you use it? *(Never to Very frequently)*
Usefulness How useful would the above product be to you or a member of your household? *(Not at all useful, to Extremely useful)*

NB. Text in parentheses represents the anchor points of the Likert scale.

On the next page, participants responded to three questions. They were presented with a list of sources (Friends/ peers/ family; news media [TV/radio/newspaper/news websites]; social media [Twitter/Facebook/Instagram/ Mumsnet/YouTube etc. and user review websites]; retailer/manufacturer websites and consumer group/Government) and asked to rate the likelihood of (a) consulting the sources if they wanted to know more information about the product and (b/c) communicating with the sources if heard about/experienced a safety related issue concerning the product, measured on a scale from ‘Not at all likely’ to ‘Extremely likely’ (see Table 5 for precise options)*.* Participants were finally asked to give an overall hazard rating for each product (“How hazardous do you consider this product to be?” rated on a 7 point scale, from ‘Not at all hazardous’ to ‘Extremely hazardous’^66^.

**Table 5:** Additional source questions – Study 1.

**Sources of information** If you wanted to know more information about [product], please rate how likely you would be to consult the following sources…. *(Not at all likely to Extremely likely)* - Friends/peers/family - News media (TV/radio/newspaper/news websites) - Social media (Twitter/Facebook/Instagram/Mumsnet/YouTube etc.) and user review websites - Retailer/Manufacturer websites - Government/Consumer group (e.g., Which?)
**Communication of information (a)** If you heard about or experienced a safety issue concerning [product], please rate how likely you would be communicate this to the following…. *(Not at all likely to Extremely likely)* - Friends/peers/family - Social media (Twitter/Facebook/Instagram/Mumsnet/YouTube etc.)/User review websites
**Communication of information (b)** If you experienced a safety issue concerning [product], please rate how likely you would be communicate this to the following…. *(Not at all likely to Extremely likely)* - News media (TV/radio/newspaper/news websites)//Retailer/Manufacturer websites//Government/Consumer group websites (e.g. Which?)

After rating all nine products, and giving ratings on sources and the genral hazard question, participants completed the General Risk Propensity Scale^20^– an eight-item scale of risk propensity (see Table 6) as well as a shortened, amended version of the Cultural Cognition Worldview Scale^68^ (see Table 7). Finally, participants were thanked, debriefed, and given a code to claim their payment.

**Table 6:** General risk propensity scale items.

General Scale Risk Propensity (shortened)
1. Taking risks makes life more fun
2. My friends would say that I'm a risk taker
3. I enjoy taking risks in most aspects of my life
4. I would take a risk even if it meant I might get hurt
5. Taking risks is an important part of my life
6. I commonly make risky decisions
7. I am a believer of taking chances
8. I am attracted, rather than scared, by risk

**Table 7:** Cultural cognition worldview scale items.

Shortened Cultural Cognition Worldview Scale
**Group or individualism-communitarianism (reverse code “C” items)** People in our society often disagree about how far to let individuals go in making decisions for themselves. How strongly you agree or disagree with each of these statements? *[strongly disagree, moderately disagree, slightly disagree, slightly agree, moderately agree, strongly agree]* 1. IINTRSTS. The government interferes far too much in our everyday lives 2. CHARM. Sometimes government needs to make laws that keep people from hurting themselves 3. IPROTECT. It's not the government's business to try to protect people from themselves 4. IPRIVACY. The government should stop telling people how to live their lives 5. CPROTECT. The government should do more to advance society's goals, even if that means limiting the freedom and choices of individuals 6. CLIMCHOI. Government should put limits on the choices individuals can make so they don't get in the way of what's good for society
**Grid or hierarchy-egalitarianism (reverse code “E” items)** People in our society often disagree about issues of equality and discrimination. How strongly you agree or disagree with each of these statements? *[strongly disagree, moderately disagree, slightly disagree, slightly agree, moderately agree, strongly agree]* 8. HEQUAL. We have gone too far in pushing equal rights in this country 9. EWEALTH. Our society would be better off if the distribution of wealth was more equal 10. ERADEQ. We need to dramatically reduce inequalities between the rich and the poor, white people and people of color, and men and women 11. EDISCRIM. Discrimination against minorities is still a very serious problem in our society 12. HREVDIS2. It seems like black people, women, LGBTQ + people and other groups don't want equal rights, they want special rights just for them 13. HFEMININ. Society as a whole has become too soft and feminine

#### Study 2

Participants were asked to imagine that they were considering purchasing a product at a fictional retailer, either online or in a high street shop. After reading the product purchase scenario, they rated the product on five characteristics, using a 7-point Likert scale (as used previously in Refs.^58,59^). The complete list of characteristics and response scales can be found in Table 8. To reduce the likelihood of participants simply clicking through without reading the questions, the next page button only appeared after 25 s. On the next page, they were informed that the product had caused harm (either as a result of non-compliance [i.e. not compliant with the safety and standards set by the regulator] or the user’s actions), based on harms identified in existing ‘Product Safety Reports’^69^ and ‘Product Recalls and Safety Notices’^70^ (see Table 3). The harm was stated as having been encountered by an external other, or friend, and then the participant was asked to re-rate the product on the five characteristics. Following this, they gave responsibility judgements for each agent and indicated how likely they would be to communicate this risk to various recipients (see Table 9).

**Table 8:** Product characteristics in Study 2 – examples for TOUR-X electric scooter.

Characteristics
Benefits How great are the benefits associated with the Tour-X Electric Scooter to you personally? *(No benefits at all, to Very great benefits)*
Severity How *severely* (i.e. degree, extent or magnitude) might you, or anyone else, be injured by the Tour-X Electric Scooter ? *(Not at all severely to Extremely severely)*
Worry How worried are you about potential risks associated with use of the Tour-X Electric Scooter? *(Not worried at all, to Extremely worried)*
Likelihood of use If you were to buy the Tour-X Electric Scooter, how likely would you be to use it? (*Not at all likely to use to Extremely likely to use)*
Hazardousness How hazardous do you consider the Tour-X Electric Scooter to be? (*Not at all hazardous to Extremely hazardous*)

**Table 9:** Post harm information questions in Study 2 – examples for Tour-X electric scooter.

**(PREFACE) Having now learned of the above risk(s) associated with the Tour-X Electric Scooter, please answer the following questions:**
Benefits How great are the benefits associated with the Tour-X Electric Scooter to you personally? *(No benefits at all, to Very great benefits)*
Severity How *severely* (i.e. degree, extent or magnitude) might you, or anyone else, be injured by the Tour-X Electric Scooter? *(Not at all severely to Extremely severely)*
Worry How worried are you about potential risks associated with use of the Tour-X Electric Scooter? *(Not worried at all, to Extremely worried)*
Likelihood of use Having purchased the Tour-X Electric Scooter, how likely would you be to use it? (*Not at all likely to use to Extremely likely to use*)
Hazardousness How hazardous do you consider Tour-X Electric Scooter to be? (*Not at all hazardous to Extremely hazardous*)
Responsibility Please indicate the extent to which it was the responsibility of the following parties to protect the individual from harm associated with the Tour-X Electric Scooter: (*0–100, Not at all their responsibility to Completely their responsibility*) *-* The product user - The retailer - The product manufacturer - The government regulator
Communication of information Please rate how likely you would be communicate this risk information to the following: (*0–100, Not at all likely to Extremely likely*) - Friends/peers/family - Social media (Twitter/Facebook/Instagram/Mumsnet/YouTube etc.)/User review websites - News media (TV/radio/newspaper/news websites) - Retailer - Product manufacturer - Government regulator - Consumer group (e.g. Which?)

Finally participants completed the General Risk Propensity Scale^20^– an eight-item scale of risk propensity (see Table 6), as well as a shortened, amended version of the Cultural Cognition Worldview Scale^68^ (see Table 7). Participants were then thanked, debriefed, and given a code to claim their payment.

### Supplementary Information

Supplementary Information.

## Data Availability

All materials and raw data are available at https://osf.io/jskg3/?view_only=424a15c7cc1a493d9ba2975d3704ec9d.
