# Supplementary material for: An integrative framework for mapping the psychological landscape of risk perception
Source: Sci Rep. 2024 May 14;14:10989. doi: 10.1038/s41598-024-59189-y (PMC11093976; doi:10.1038/s41598-024-59189-y)
Supplement: Supplementary file 1 — Supplementary Information. [file 41598_2024_59189_MOESM1_ESM.docx]

| **Supplementary Materials 1**  *Table S1. Products and Associated Descriptions – Study 1 and 1B*  Note: Descriptions were developed using the Delphi method (OPSS regulators and experts) and reference to existing research. |
| --- |
| **Products** |
| **Arcing lighter**  A flameless, battery powered electronic lighter, which produces heat using a small arc of high-voltage electrical current. |
| **At home teeth whiteners**  Teeth whitening done at home by the individual, using a kit to bleach teeth to make them lighter. |
| **Baby car seat**  A rearward-facing portable seat, secured to a car seat, with straps to buckle a baby in, used to transport babies between birth and 15 months. |
| **Balcony BBQ**  Used on a balcony or roof terrace, a grill for cooking food outdoors, using either gas or hot coals. |
| **Bicycle (non-electric)**  A human powered device, consisting of two wheels attached to a frame, handlebars, a saddle and pedals, used for personal transport. |
| **Blind with looped cords**  A window covering used for blocking light, which is operated using a pull-cord or chain. |
| **Button battery**  A small, single cell battery which is round and flat, typically used in watches, toys, hearing aids, car keys and other small devices. |
| **Carbon monoxide detector**  Devices which monitor and measure levels of carbon monoxide in the air, sounding an alarm if it detects the presence of carbon monoxide. |
| **CBD health products**  CBD (cannabidiol) is a chemical found within hemp and cannabis. CBD health products include nutritional supplements and cosmetic products (e.g., moisturisers, shampoo), used for health and beauty purposes. |
| **Children's clothing with cords and drawstrings**  Items of children's clothing (up to age 14) which contain cords or drawstrings to adjust or fasten something. Examples include those used in sweatshirt hoods, shorts and halter-necks. |
| **Children's fancy dress**  Costumes specifically designed for children to dress up in, often made from polyester and/or nylon. |
| **Christmas tree lights**  Lights used to decorate Christmas trees, which are powered by battery or mains electricity. |
| **Dishwasher**  An electrical appliance used to clean dishware and cutlery automatically, using hot water. |
| **E-cigarettes**  An electronic, battery powered device which is used to simulate the experience of smoking by heating a liquid into a vaporised solution, which the user inhales and exhales. This solution typically contains nicotine, flavourings and other chemicals. |
| **Electric iron**  An electrical appliance, which uses heat to press folds out of clothes. |
| **Electric kettle**  An electrical appliance, which uses a heating element to boil water. |
| **Electric pool heater for paddling pools**  A heating tank which brings cold water in and pumps warm water back into the pool, powered by electricity and used in paddling pools. |
| **Electric scooter**  A device consisting of two or three wheels, handlebars and a floorboard which is stood on while riding, powered by an electric motor. Used for personal transport. |
| **Fireplace surround**  An object which sticks out from the outer wall of a fireplace and surrounds the fireplace opening, typically made up of the sides and mantel. |
| **Fireworks (personal use)**  Low explosive pyrotechnic devices, which may be used for aesthetics, entertainment purposes and/or religious celebrations/ceremonies at one's home. |
| **Fridge/freezer**  An electrical appliance which comprises of a self-contained refrigerator and freezer, artificially kept cool to store food and drink. |
| **Hair clippers**  An electric tool used to cut human hair, featuring a motor, blade and guards, the latter of which attaches to the top of the blade. Guard size dictates how much hair is cut off. |
| **Hair straighteners**  An electric tool featuring two heated metal or ceramic plates, which are guided through the hair to straighten it. |
| **Hair dye**  Chemicals which are used to change the colour of human hair, either temporarily or permanently. |
| **Hand sanitiser**  A liquid or gel, typically containing alcohol and applied to the hands to kill viruses and bacteria. |
| **Inclined baby sleeper**  A freestanding, portable product with an inclined sleep surface, allowing babies to sleep at an angle between 10 and 30 degrees. These are sometimes called loungers, rockers or nappers. |
| **Ladder**  A piece of equipment used to reach high places, consisting of two long pieces of wood or metal, joined together by horizontal rungs. |
| **Laser pointer**  A small, pen-like device which uses a power source (typically a battery) and diode laser to produce an intense beam of visible, monochromatic light, usually to highlight a point of interest. |
| **Laundry detergent**  A cleaning agent used for washing dirty laundry, which comes in either powder or liquid form. |
| **Microwave oven**  An electric oven which uses high-frequency electromagnetic waves to cook food. |
| **Musical greeting card**  A greetings card which plays music when it is opened. Such cards typically contain a small device embedded in the card, powered by a small button battery. |
| **Neodymium magnets in construction toys**  Super strong, small spherical magnets, which can be separated and put together into various shapes and patterns**.** |
| **Outdoor trampoline (personal use)**  A piece of equipment consisting of a piece of taut, strong fabric stretched between a frame using coiled springs, which is jumped upon. Refers to those used at one’s home for exercise or recreation. |
| **Oven**  An appliance for cooking food using heat. |
| **Pikler triangle climbing frame**  A wooden indoor climbing structure for babies and toddlers, designed to encourage the practise of motor development skills. |
| **Power hedge trimmer**  A gardening tool, consisting of a motor powered by gas, electricity or battery and a long, toothed blade, used to cut hedges and bushes. |
| **Power mower**  A motor driven lawn mower which is powered by gas or electric and used to cut grass. |
| **Printer cartridge**  A cartridge containing liquid ink which is inserted into an inkjet printer and used for printing. |
| **Recliner chair**  An armchair or sofa which has an adjustable back and footrest to allow the sitter to recline in it, either using a manual or electrical mechanism. |
| **Rug**  A floor covering, typically used for decorative purposes, consisting of a piece of thick, shaped fabric, not extending over the entire floor. |
| **Scatter cushion**  A small cushion, typically used for decorative purposes, consisting of a cover and a filling. Some scatter cushions have a removable cover and insert, accessed through a zipped or buttoned opening. |
| **Smart doorbell**  An internet-connected doorbell linked to a smartphone or electronic device which notifies the owner when a visitor arrives at the door. |
| **Smart light bulb converter**  A connector for changing a light bulb into a smart bulb, which can be linked to a smartphone or electronic device for remote operation. |
| **Sparklers**  A type of hand-held firework used for entertainment, made from a metal wire which is coated at the end with a flammable substance which, when lit, emits sparks as it burns. |
| **Above ground swimming pool**  A freestanding water retaining structure which sits on top of the ground, used for leisure and exercise purposes. |
| **Talc-based cosmetic products**  Talcum powder is a naturally occurring mineral, mined from the earth and used as an ingredient in many cosmetic products, such as make-up and baby powder. |
| **Travel adaptor**  A connector which changes the plug shape to match the electrical outlet. This allows use of equipment which cannot be directly inserted into the standard plug. |
| **Treadmill**  An exercise machine, consisting of a continuous moving belt used to walk or run on. |
| **Tumble dryer**  An electrical appliance which dries wet laundry using warm air. |
| **USB charging plug/cable (purchased separately)**  USB wall charging plugs or cables, purchased separately from the device they are intended to power/charge. |
| **UV light sanitising wand**  A handheld device containing lights which emit ultra-violet rays to kill/inactivate viruses and bacteria. |
| **Virtual reality headset**  A head-mounted device which covers the eyes and allows the user to interact with simulated environments. |
| **Washing machine**  An electrical appliance used to wash laundry using water and detergent. |
| **Weedkiller (herbicide)**  A chemical substance, used to kill or inhibit the growth of unwanted plants. |

**Supplementary Materials 2**

**Data Preparation**

*Study 1*: Although a ‘don’t know’ option was not included in the study (following Fife-Schaw and Rowe’s [^66^] recommendation for participants to avoid its use as much as possible), participants were able to skip questions. As per our pre-registered analysis plan, we checked to see if any of the products had >15% missing responses, though this was not the case. Where there were missing values on any of the 11 characteristics to be featured in the principal component analysis (Table S2), these were replaced by the mean value (as in ^67,68^). For the rest of the variables, cases with missing values were removed from the following analyses, unless otherwise stated.

Contrary to our pre-registration, we did not recode the benefit, familiarity, known to those exposed, likelihood of use and perceived usefulness items to aid interpretation of the results. Importantly, the PCA solution does not differ if these are recoded.

Risk propensity and grid worldview scores were positively skewed and thus we used a square root transformation to achieve an approximately normal distribution. Risk propensity, grid and group worldview scores were all subsequently scaled. This was also the case for Study 2.

Ratings for three of the overarching product categories were skewed and thus we transformed these to achieve approximately normal distributions. For the product age and power/fuel ratings, we used the transformation (-1*(log(100-x). For the vulnerable groups ratings, we used a log transformation. We then subsequently scaled all of the product category ratings.

We created two new communication variables, ‘*communication – personal*’ – averaging seeking/sharing information responses across ‘friends/peers/family’ and ‘social media/user review websites’, and ‘*communication – impersonal* – averaging responses across ‘news media’, ‘retailer’, ‘product manufacturer’, ‘government regulator’ and ‘consumer group’.

Analysis presented is based on the data aggregated across individuals, to identify differences amongst products. This analysis differed slightly to what was originally pre-registered, in that we adopted a more general and conservative approach by (a) performing SEM rather than conducting separate models for each response variable and (b) including random slopes in the models. Also, given the multicollinearity associated with hazardousness, we were not able to include this as a fixed effect.

*Study 2:* We summed together worry, severity and hazardousness ratings at each time point to create two measures of *dread.* Similarly, we summed benefits and likelihood of use ratings to create two measures of *benefits.* These were then scaled by dividing by the SD.

We created a new ‘*external responsibility*’ variable, averaging responses across each responsibility rating given for retailer, manufacturer and government regulator.

We created two new communication variables: ‘*communication – personal*’ averaging sharing information responses across ‘friends/peers/family’ and ‘social media/user review websites’, and ‘*communication – impersonal* – averaging responses across ‘news media’, ‘retailer’, ‘product manufacturer’, ‘government regulator’ and ‘consumer group’.

**Supplementary Materials 3**

**Principal Components Analysis – Study 1**

We created a correlation matrix, featuring all of the variables in the principal components analysis (PCA), in order to check that all of the variables had at least one correlation where *r* ≥ 0.3 ^69^. All of the variables did and thus were retained for the primary analysis.

We carried out a PCA of the aggregated data, using a Varimax rotation. Such a rotation results in more interpretable clusters of factors ^70^, and has been used in previous psychometric studies ^68^ including those focusing on consumer products ^61^ as well as in the original study employing the psychometric paradigm ^55^. Three components were retained based on our pre-registered criteria <https://osf.io/jskg3/?view_only=424a15c7cc1a493d9ba2975d3704ec9d>.

**Table S2. Loadings from the PCA – Aggregated Data.**

|  | *Benefits (37.0%)* | *Dread (29.3%)* | *Responsibility (26.7%)* |
| --- | --- | --- | --- |
| Benefits | ***0.973*** |  |  |
| Familiarity | ***0.816*** |  |  |
| Likelihood of use | ***0.904*** |  |  |
| Usefulness | ***0.971*** |  |  |
| Severity |  | ***0.953*** |  |
| Likelihood of injury |  | ***0.916*** |  |
| Worry | *-0.465* | ***0.854*** |  |
| Known to those at risk | *0.439* | ***0.635*** | *0.586* |
| Control | *0.440* |  | ***0.790*** |
| Blame |  |  | ***-0.949*** |
| Responsibility for protection |  |  | ***-0.932*** |

Note: Where characteristics cross-loaded on more than one component, we selected the component in which the characteristic had the highest loading. Items in bold represent the final solution.

Figure S1 gives an overview of the relative differences in perceptions for each product, according to the benefits and dread components.

*
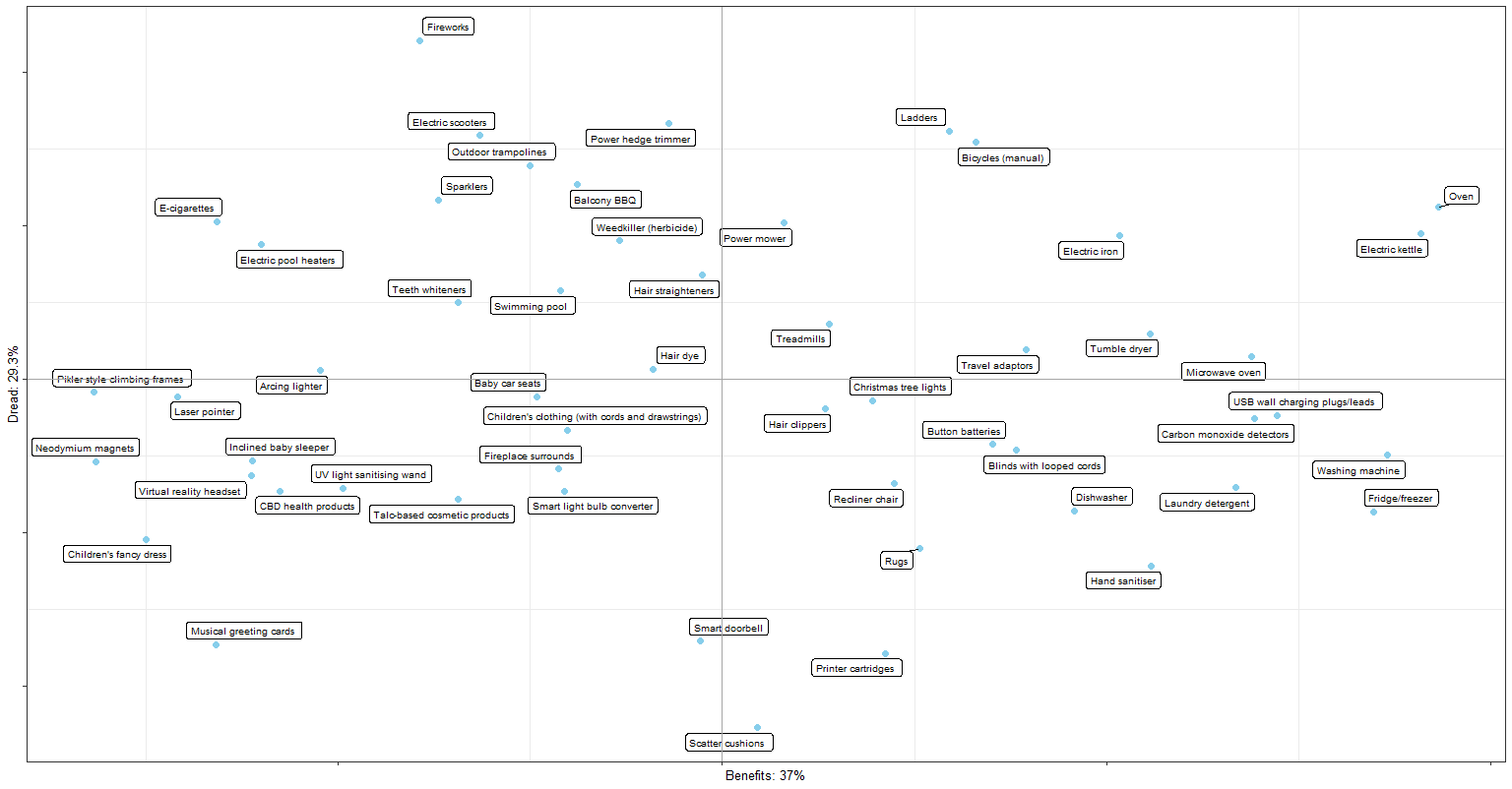
Figure S1. Location Of Products Within the Benefits and Dread Dimension Space.*

**Supplementary Materials 4**

**Model Specification Process**

***Study 1***

*(Risk Perceptions/Risk Tolerance plus Risk Communication)*: We specified a weakly informative prior ~ *N* (0,10) for all model parameters. This prior specification was used as no existing similar data existed. Four Markov chains were implemented for each parameter and the model was run for 25,000 iterations with a burn-in phase of 5000 iterations. To assess chain convergence, the Gelman and Rubin ^71^ convergence diagnostic was implemented with the default setting. The Gelman and Rubin diagnostic indicated that convergence was achieved. Trace plots for a random selection of model parameters were subsequently checked, and further supported the claim that convergence was obtained.

***Study 2***

We specified a weakly informative prior ~ *N* (0,10) for all model parameters. This prior specification was used as no existing similar data existed. Four Markov chains were implemented for each parameter and the model was run for 25,000 iterations, with a fixed number of post burn-in iterations of 5000. Residual correlations were not fitted. To assess chain convergence, the Gelman and Rubin ^71^ convergence diagnostic was implemented with the default setting and indicated that convergence was achieved. Trace plots for a random selection of parameters were subsequently checked, which further supported the claim that convergence was obtained.

**Supplementary Materials 5**

**Study 1B**

In a follow-up study to Study 1, we investigated how individuals perceive consumer products according to four characteristics identified from previous research as potentially influential in shaping risk perceptions (Study 1B). That is – the (relative) age of the product; its main purpose (e.g., leisure versus household goods); whether it involves power or fuel and whether vulnerable groups (such as children or the elderly) particularly use the product.

***Participants***

A sample of 382 participants were recruited from Prolific Academic ([www.prolific.ac](http://www.prolific.ac)). They were required to be over 18, in order to meet ethical requirements and to not have completed any of our previous consumer risk perception studies. Participants were excluded if their responses for any of the products had a SD < 0.5, leaving a final sample of 371 participants (50.9% male, 48.0% female, 1.1% other or prefer not to say). Participants were paid £0.85 for the study, which took around 8-10 minutes to complete.

***Questionnaire***

The fifty-four products featured in Study 1 were included in the study, presented with the same short descriptive sentences. Participants were randomly presented with nine of the fifty-four products to rate. Each product was rated on a series of four characteristics, using a 0 to 100 slider. The complete list of characteristics and questions can be found in Table S3. To reduce the likelihood of participants simply clicking through without reading the questions, the next page button only appeared after seven seconds.Table S3. Over-arching Product Categories.

| **Characteristic** |
| --- |
| **Product Age**  To what extent do you agree that the above is a: an old, established product (versus a new product)? *(Completely disagree the above is old to Completely agree the above is old)* |
| **Main Purpose**  To what extent do you agree that the above is designed as a: leisure, recreation or personal care product (versus a household good, appliance or healthcare product)?  *(Completely disagree the above is designed as a leisure, recreation or personal care product to Completely agree the above is designed as a leisure, recreation or personal care product).* |
| **Power/Fuel**  Does the above product involve electricity, batteries, fire or chemicals?  *(Does not involve electricity, batteries, fire or chemicals at all to Involves electricity, batteries, fire or chemicals)* |
| **Vulnerable Groups**  To what extent do you agree that vulnerable groups (such as children or the elderly) use or interact with the above product more than other groups of people? (*Completely disagree vulnerable groups use or interact with the above more than other groups to Completely agree vulnerable groups use or interact with the above more than other groups)* |

***Procedure***

The study was run using Qualtrics. Before beginning the main task, participants were asked a series of demographic questions, as specified in Study 1. They were also asked for their Prolific ID, used for payment and completed a captcha question.

Firstly, participants were presented with instructions for the task. Participants were then presented with one of the products and asked to rate the product on four characteristics, using a 0-100 slider (see Table S3). Both products and characteristics were presented in a random order, to control for order effects. The subsequent screen showed another randomly presented product, and so on and so forth, until the participant had rated nine of the products. Finally, participants were thanked, debriefed, and given a code to claim their payment.

**Supplementary Materials 6**

**Product Clustering**

As per our pre-registration, in Study 1, we used the UMAP method ^39^ to identify whether products clustered within the risk perception space, both generally and in relation to four over-arching product categories: (relative) *age* of the product; its main *purpose* (e.g., leisure versus household goods); whether it involves *power or fuel* and whether *vulnerable groups* (such as children or the elderly) particularly use the product. Product ratings according to each of these categories were obtained from a separate study, see Supplementary Materials 2.

There was some evidence to suggest that products generally clustered within the risk perception space, with three clusters seen in Figure S2. The top right cluster relates to what we call household goods, with the left hand and bottom clusters relating more to leisure goods. We saw some evidence of clustering by age, with the majority of newer products grouping together but limited evidence was seen for clustering by power or by vulnerable groups.

*Figure S2.
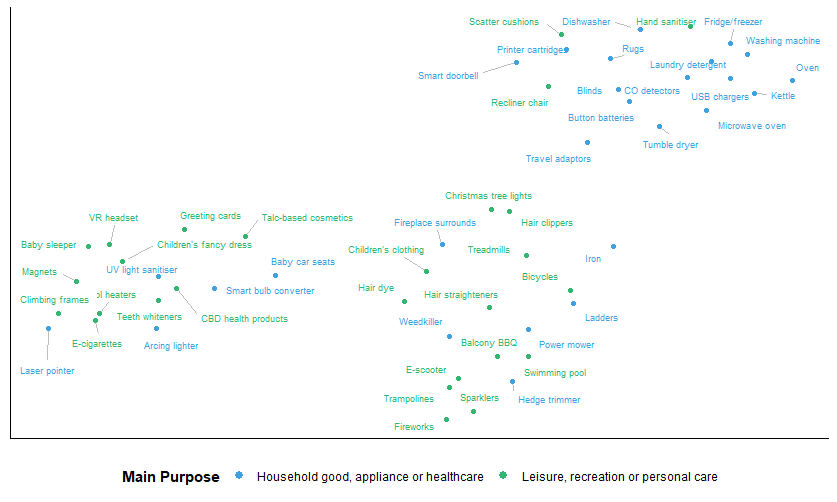
UMAP product clustering by main purpose – Study 1.*

**Supplementary Materials 7**

*Study 2 – Results of the full SEM predicting: Dread, Benefits (Time 2), Communication and Responsibility Attributions.*

*Figure S3. Clear (≠ 0) predictors of Dread, Benefits (T2), Communication and Responsibility Attributions – Study 2. Estimates were derived from the final model specifications: Dread, Benefits ~ ((Product + Place of Purchase + Harm Cause + Harm Experience)^2) + (Age + Gender + Children + Risk Propensity + Grid Worldview + Group Worldview) * (Place of Purchase + Harm Cause + Harm Experience)).
Communication – Personal/Impersonal, Responsibility – User/External ~ ((Product + Place of Purchase + Harm Cause + Harm Experience)^2) + (Dread + Benefits) + (Age + Gender + Children + Risk Propensity + Grid Worldview + Group Worldview) * (Place of Purchase + Harm Cause + Harm Experience))*

*
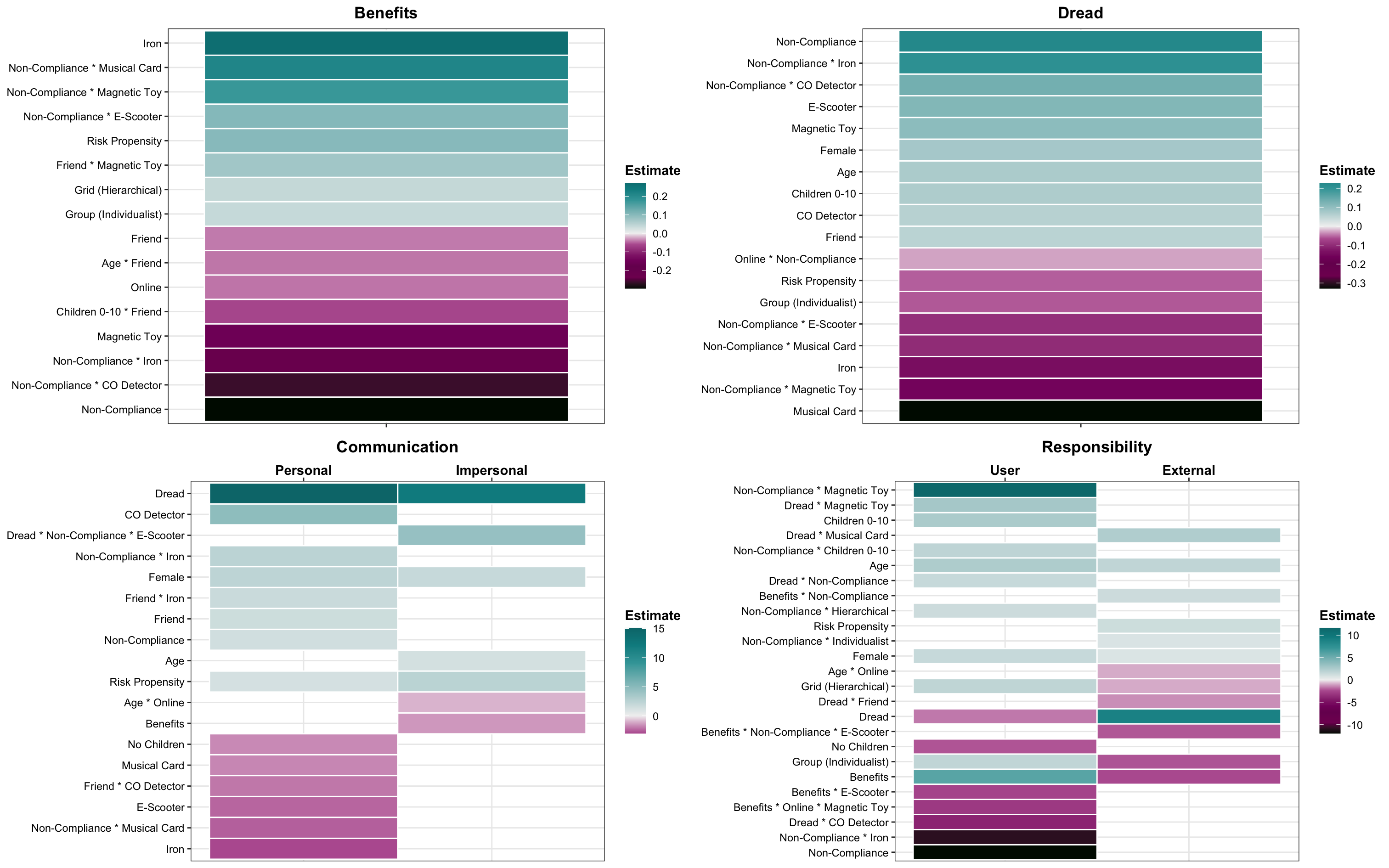
*

Note: Higher estimates indicate high benefits, dread, increased likelihood of communicating risk information and increased responsibility.
